# Retraction Note: Warming accelerates global drought severity

**DOI:** 10.1038/s41586-026-11027-z

**Published:** 2026-09-02

**Authors:** Solomon H. Gebrechorkos, Justin Sheffield, Sergio M. Vicente-Serrano, Chris Funk, Diego G. Miralles, Jian Peng, Ellen Dyer, Joshua Talib, Hylke E. Beck, Michael B. Singer, Simon J. Dadson

**Affiliations:** 1 School of Geography and the Environment, University of Oxford, Oxford, UK; 2 School of Geography and Environmental Science, University of Southampton, Southampton, UK; 3 Instituto Pirenaico de Ecología, Consejo Superior de Investigaciones Científicas (IPE-CSIC), Zaragoza, Spain; 4 Santa Barbara Climate Hazards Center, University of California, Santa Barbara, CA USA; 5 Hydro-Climatic Extremes Lab (H-CEL), Ghent University, Ghent, Belgium; 6 Department of Remote Sensing, Helmholtz Centre for Environmental Research - UFZ, Leipzig, Germany; 7 Institute for Earth System Science and Remote Sensing, Leipzig University, Leipzig, Germany; 8 UK Centre for Ecology and Hydrology, Wallingford, UK; 9 Climate and Livability Initiative, King Abdullah University of Science and Technology, Thuwal, Saudi Arabia; 10 School of Earth and Environmental Sciences, Cardiff University, Cardiff, UK; 11 Earth Research Institute, University of California, Santa Barbara, CA USA; 12 Water Research Institute, Cardiff University, Cardiff, UK

**Keywords:** Hydrology, Hydrology, Climate sciences

Retraction to: *Nature* https://doi.org/10.1038/s41586-025-09047-2 Published online 4 June 2025

The authors have retracted this article after being notified of errors in the paper and independently identifying further issues. First, area-weighted grid cells were not used when calculating study-wide averages, an error that affected numerical values in the main text, Fig. 1b, and Extended Data Figs. 4 and 8. Second, results based on GLEAM v3 instead of GLEAM4 were inadvertently used in Extended Data Fig. 4: the two datasets are largely consistent for 1981–2021, but the area weighting based on GLEAM4 resulted in a reduced drought-affected area in 2022. Third, the underlying code included a timestepping error in the computation of regional drought-affected areas, meaning that dates were not properly parsed when subsetting the data for the 1981–2017 and 2018–2022 periods. This misclassification of time steps affected the regional averages in Extended Data Fig. 8h. Finally, it was not clearly explained how masking was applied in calculations. To clarify this point: the arid regions mask (defined as areas with mean annual precipitation < 180 mm) was applied to the SPEI time series in Fig. 1a but not Fig. 1b (the same distinction applies to Extended Data Figs. 4 and 8). The rationale for the masking approach was that in dry regions, extremely negative SPEI is common (associated with zero precipitation), and it was judged that their inclusion would have generated unrealistically low average values. Because the % values in Fig. 1b are insensitive to negative outliers, the mask was not applied there. Although the authors are willing to correct and clarify the above matters, given the scope of the necessary corrections and the need for additional revision and peer review, the authors have decided to retract the paper and to resubmit a fully revised and corrected version for peer review. All authors agree with the retraction.

